# Improving PCASL at ultra‐high field using a VERSE‐guided parallel transmission strategy

**DOI:** 10.1002/mrm.28173

**Published:** 2020-01-23

**Authors:** Yan Tong, Peter Jezzard, Thomas W. Okell, William T. Clarke

**Affiliations:** ^1^ Wellcome Centre for Integrative Neuroimaging FMRIB Division Nuffield Department of Clinical Neurosciences University of Oxford United Kingdom

**Keywords:** parallel transmission (pTx), perfusion, pseudo‐continuous arterial spin labeling, RF shimming, ultra‐high field

## Abstract

**Purpose:**

To improve the labeling efficiency of pseudo‐continuous arterial spin labeling (PCASL) at 7T using parallel transmission (pTx).

**Methods:**

Five healthy subjects were scanned on an 8‐channel‐transmit 7T human MRI scanner. Time‐of‐flight (TOF) angiography was acquired to identify regions of interest (ROIs) around the 4 major feeding arteries to the brain, and $B_{1}^{+}$ and B_0_ maps were acquired in the labeling plane for tagging pulse design. Complex weights of the labeling pulses for each of the 8 transmit channels were calculated to produce a homogenous radiofrequency (RF) ‐shimmed labeling across the ROIs. Variable‐Rate Selective Excitation (VERSE) pulses were also implemented as a part of the labeling pulse train. Whole‐brain perfusion‐weighted images were acquired under conditions of RF shimming, VERSE with RF shimming, and standard circularly polarized (CP) mode. The same subjects were scanned on a 3T scanner for comparison.

**Results:**

In simulation, VERSE with RF shimming improved the flip‐angles across the ROIs in the labeling plane by 90% compared with CP mode. VERSE with RF shimming improved the temporal signal‐to‐noise ratio by 375% compared with CP mode, but did not outperform a matched 3T sequence with a matched flip‐angle.

**Conclusion:**

We have demonstrated improved PCASL tagging at 7T using VERSE with RF shimming on a commercial head coil under conservative SAR limits at 7T. However, improvements of 7T over 3T may require strategies with less conservative SAR restrictions.

## 1 INTRODUCTION

Arterial spin labeling (ASL) is a noninvasive perfusion imaging technique which offers various clinical applications in areas such as stroke, dementia, and chronic vascular diseases.^1^ During an ASL scan, spins in blood vessels upstream of the imaging plane are inverted or saturated (labeled). After a postlabeling delay to allow the labeled blood water to reach the tissue, an image is acquired, for example with an echo planar imaging (EPI) readout.^2^ ASL labeling techniques can be divided into 3 categories: continuous ASL (CASL), pulsed ASL (PASL), and pseudo‐continuous ASL (PCASL). Among the 3 approaches, PCASL was recommended by the ISMRM Perfusion Study Group and European Consortium for ASL in Dementia due to its relatively high signal‐to‐noise ratio (SNR) and clinical applicability.^1^ For brain imaging using PCASL, blood is typically labeled in the 4 main feeding arteries: the right internal carotid artery (RICA), right vertebral artery (RVA), left internal carotid artery (LICA), and left vertebral artery (LVA).

ASL has intrinsically low SNR due to the relatively low amount of perturbed water magnetization delivered by means of blood flow versus the signal from static tissue. Thus, ASL can benefit from ultra‐high field (>3T, UHF) strengths due to the increase in overall SNR, and the increase of blood longitudinal relaxation time (*T*_1_).^3^ These benefits of 7T have been used to explore ASL applications that are challenging at lower field strength, such as to measure perfusion signal from white matter^4^ and to enable investigation of the subtle metabolic consequences of mitochondrial DNA mutations.^5^

Despite these theoretical advantages, the implementation of ASL at UHF is not trivial.^3^ An increase of $B_{1}^{+}$ and B_0_ inhomogeneity and reduced $B_{1}^{+}$ efficiency below the brain at higher field strength degrade the performance of the labeling radiofrequency (RF) pulse(s), leading to poor labeling efficiency which, in turn, diminishes the perfusion signal.^3^ Also, for a given $B_{1}^{+}$ field strength, the specific absorption rate (SAR) scales roughly quadratically with B_0_ field strength, reducing the permitted labeling pulse train duration (for PCASL) and/or amplitude. Various strategies have been proposed to address the $B_{1}^{+}$ challenges of ASL at UHF. Zuo et al placed the labeling plane at the level of the base of the cerebrum due to deteriorating $B_{1}^{+}$ and B_0_ inhomogeneity in more inferior positions, but this approach limits the size of the imaging volume.^6^ Wang et al used dielectric pads to improve $B_{1}^{+}$ efficiency of the tagging pulses.^7^ Stafford et al introduced an actively decoupled dual transceiver coil system which consists of an 8‐channel transceiver head coil and a 3‐channel transceiver labeling coil.^8^ Zimmer et al^9^ presented a $B_{1}^{+}$‐optimized adiabatic inversion pulse in conjunction with the “PICORE” labeling scheme^10^ to improve the perfusion signal.

At UHF, among the labeling strategies, CASL has limited applicability because RF amplifiers in most clinical scanners cannot produce a continuous RF pulse.^11^ A head‐only transmit coil is often used to image the brain at UHF due to challenges in designing whole‐body transmit coils at UHF. As a result, PASL approaches are somewhat limited, because a large region that extends further away from the brain is ideally required to be labeled, where $B_{1}^{+}$ efficiency decreases significantly. Thus, PCASL is particularly appealing at UHF. However, PCASL is sensitive to $B_{1}^{+}$ and B_0_ inhomogeneity, which reduce its efficiency, and by SAR restrictions that limit the ability to use a larger flip‐angle (FA) at higher field strengths. These issues are addressed by the proposed method in this work.

Parallel transmission (pTx) provides additional degrees of freedom that allows for constructive and destructive interference of different RF transmit channels to mitigate the $B_{1}^{+}$ inhomogeneity. RF shimming (or “static pTx”) aims to produce a more homogeneous and more accurate FA distribution within the labeling region of interest (ROI) by adjusting the complex weights (phase and amplitude) of each available RF channel. To mitigate the challenges of SAR constraints a Variable‐Rate Selective Excitation (VERSE) RF pulse approach^12^ was used in this study, without compromising the PCASL inter‐pulse duration or slice profile. VERSE is a re‐fabrication technique rather than a pulse design technique, because it calculates a new pulse and a time‐varying gradient waveform from a previously designed pulse.^12^ In this study, we present a VERSE‐guided RF shimming strategy to further improve PCASL labeling under explicit power constraints. This study builds on work presented in a previous conference proceeding showing that RF shimming improves the labeling efficiency of PCASL at 7T.^13^

## 2 METHODS

Five subjects (male; mean age, 33.8 years; range, 27 to 52 years) were scanned using a Siemens Magnetom 7T scanner (Erlangen, Germany) equipped with an 8‐channel pTx capability. A Nova Medical Inc. (Wilmington MA) 8Tx32Rx head coil was used. All experiments were performed under an agreed technical development protocol approved by the Oxford University Clinical Trials and Research Governance office, in accordance with International Electrotechnical Commission and United Kingdom Health Protection Agency guidelines. To ensure subject safety, the pTx system was operated under the Siemens‐defined “protected mode”. In this mode, the 6 min and 10 s average power limit are set to 12.39 W and 24.78 W (as measured by the Power Absorption Limiter Multi Channel, PALI MC), respectively. The power limits, provided by the coil vendor (Nova Medical) in agreement with scanner vendor (Siemens), are based on a limit at the coil plug (10 W/20 W for 10 s/6 min, respectively) and include a calibration factor for cable losses.

**Figure 1:**
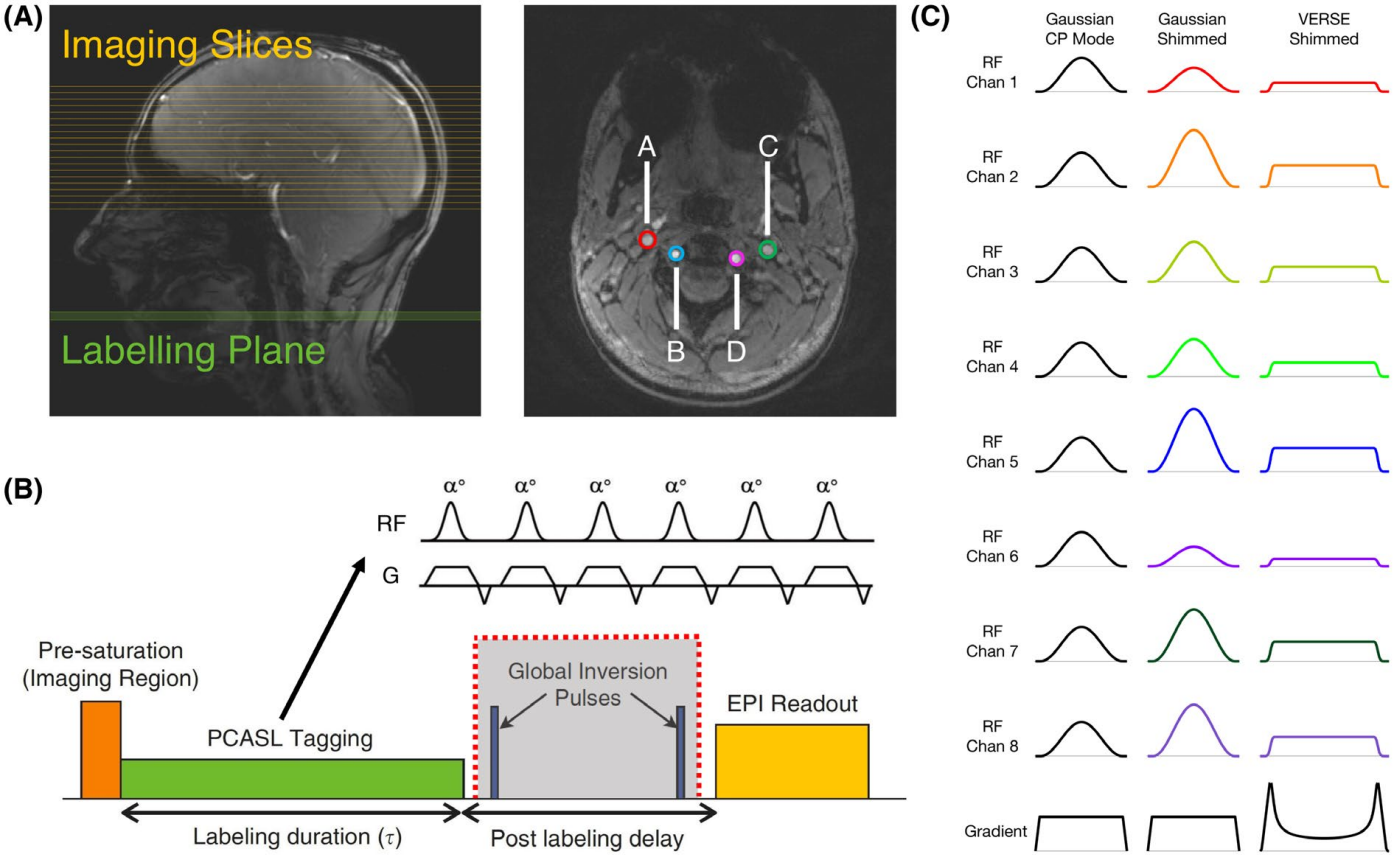
A, Labeling plane (green) and imaging slices (yellow) for PCASL on a sagittal localiser, and time‐of‐flight image of the labeling plane through the neck. The 4 main brain‐feeding arteries are circled in color: A, RICA; B, LICA; C, RVA; D, LVA. B, Schematic of the PCASL sequence. The global inversion pulses (grayed) were discarded at 7T due to SAR constraints. C, RF and gradient pulses of the 3 labeling schemes

$B_{1}^{+}$ maps were acquired using a 2D “STE first” phase‐cycled DREAM sequence.^14^, ^15^ B_0_ maps were acquired using a dual‐echo gradient‐echo sequence. A time‐of‐flight (TOF) sequence was used to select a transverse labeling plane location (though the V3 segment of the vertebral arteries) and to identify the 4 main brain‐feeding arteries within this plane (RICA, RVA, LICA, and LVA; Figure 1). Four elliptic ROIs on the labeling plane (thickness = 2.5 mm) were then manually selected around the 4 arteries from the TOF image (average size = 76.4 mm^2^). The PCASL sequence parameters were based on a study by Okell et al^16^ and are listed in Table 1. Presaturation of the imaging region was achieved by means of a 4‐pulse variable angle WET (*w*ater suppression *e*nhanced thorough *T*_1_ effects) scheme.^17^ Additional inversion pulses for background suppression^18^ were not used due to the high RF power deposition of these adiabatic pulses.

**Table 1:** 7T experiment sequence parameters

Parameter	Value
Gaussian RF pulse duration	600 μs
VERSE RF/gradient pulse duration	950 us
RF labeling pulse separation	1.2 ms
Mean tagging gradient	0.8 mT/m
Tagging gradient during Gaussian pulses	6 mT/m
Maximum VERSE gradient	12.27 mT/m
Target flip‐angle	20°
Labeling duration (τ)	1 s
Post‐labeling delay (PLD)	1 s
6‐min average RF power limit	12.39 W
10‐s average RF power limit	24.78 W
Voxel size	2 × 2 × 4 mm
Matrix size	110 × 110
Partial Fourier factor	6/8
GRAPPA factor	2
Number of slices	19
Echo time (TE)	13 ms
Volume repetition time (TR)	6 s
Measurements	32 (16 pairs)
Acquisition time	3.5 min

The RF shimming calculation was carried out on a single Gaussian or VERSE pulse, producing a single shim which was applied to the whole labeling train. The VERSE pulse was obtained using an approach proposed by Hargreaves et al with a targeted RF power reduction factor of 2,^19^ and VERSE was applied before optimization of the weights. This reduction factor permitted the inter‐pulse separation to be preserved. The labeling pulse FA optimization strategy was based on an algorithm by Dupas et al,^20^ and was formulated in the small tip angle approximation and in 2D. This is because the $B_{1}^{+}$ map resolution is relatively coarse (4 mm isotropic) and the $B_{1}^{+}$ field does not change with high spatial frequency in the vicinity of the labeling plane. The pulse calculation was performed on a MacBook Pro laptop (Apple Inc. Cupertino, CA) with a 2.7 GHz Intel Core i5 processer (Intel Corporation, Santa Clara, CA) and 16 GB memory. A spin dynamics matrix, *A*, was constructed the same way as described by Equation 1 of Dupas et al,^20^ including off‐resonance measurements taken from the B_0_ maps, transmit sensitivities taken from the $B_{1}^{+}$ maps, and each voxel’s position, and the gradient waveforms and RF pulse shape for the Gaussian and the VERSE pulse. The RF shimming problem is then presented as one that minimizes |||*Ab*|−θ||_2_, subject to explicit per‐channel and sum‐of‐channels average and absolute power limits, where *b* is the complex weight of each RF channel in Volts, and θ is the desired FA in each voxel in the ROI.^21^ The RF power limit was explicitly incorporated into the optimization algorithm. Therefore, this limit was relaxed to investigate how much gain in FA could be achieved if future vendor updates were to allow for a less conservative RF power limit. A relaxation factor of two corresponds to twice the locally adopted RF power limit being applied. Such RF power relaxations are achieved by changing the constraint of the fmincon function. The RF shims were only applied to the labeling pulses, and no pTx strategy was used to mitigate the excitation $B_{1}^{+}$ inhomogeneity in this study. The FA of the excitation pulse used in imaging was 90°.

Similar to the approach by Dupas et al, the optimization algorithm consists of 2 steps: the variable‐exchange (V‐E) method and the active‐set (A‐S) method.^20^ Because the A‐S algorithm is a nonlinear method, the starting point is crucial to the final result. Therefore, the V‐E method was used to generate candidate RF shims for the A‐S method. The Tikhonov regularization constant lambda used in the V‐E method indirectly controls for the RF power, and the cost function was adjusted to |||Ab|−θ||^2^+λ||b||^2^ for the V‐E method. To increase the probability of finding a good local minimum, the V‐E method, with an initial phase defined to be that from the CP mode $B_{1}^{+}$ phase map, was run with different Tikhonov regularization constants “λ” (λ = 10^−6^, 10^−5^, 10^−4^, 10^−3^, 10^−2^, 10^−1^) to generate candidate RF shims of distinct energy contents.^22^ The λ values were chosen heuristically to produce acceptable results in FA with a feasible RF voltage. The shims generated by the V‐E method were used to initialize the A‐S method, and the resulting shim that minimizes the cost function (|||Ab|‐θ||^2^) was chosen as the final solution. The RF power (||b||^2^/50Ω) limit was explicitly incorporated into the A‐S optimization algorithm. The optimization algorithm mentioned above was implemented in MATLAB (The MathWorks, Natick, MA).^23^ An example dataset and the MATLAB code used for the RF pulse optimization can be found at https://doi.org/10.5287/bodleian:gJRO8qpAz.

The same subjects were scanned using a Siemens Prisma 3T scanner (Erlangen, Germany) for comparison with the 7T data. Four PCASL sequences were applied during the 3T session: (1) A matched sequence with a matched labeling pulse FA (7T FA defined as the mean FA achieved in the 4 feeding vessels in the RF shimming simulation); (2) A matched sequence, but with a labeling pulse FA of 20°; (3) A matched sequence with an optimized labeling scheme (20° FA, labeling pulse duration = 500 μs, RF separation = 1000 μs, labeling duration = 1.8 s); (4) A fully optimized 3T sequence with the same scan time (20° FA, labeling pulse duration = 500 μs, RF separation = 1000 μs, labeling duration = 1.8 s, TR = 3.7 s, background suppression double inversion on). An M_0_ image was acquired at 3T and 7T for the quantification of cerebral blood flow.

Data analysis was performed using tools from the FMRIB Software Library (FSL)^24^: BET^25^ (brain extraction), MCFLIRT^26^ (motion correction), FLIRT^26^, ^27^ (linear registration), BASIL^28^ (CBF quantification) tools were used. Geometric distortion correction was performed on the 7T data due to more pronounced EPI‐induced image distortion. Motion correction was applied to the label and control images. Segmentation of gray matter, white matter, and cerebrospinal fluid (CSF) was performed on a T_1_‐weighted structural image that was also acquired. The GM partial volume was then transformed into the ASL space, and a GM mask was generated using a GM partial volume fraction threshold of 0.4. The labeling efficiency of each labeling scheme was calculated using Bloch simulation of the labeling pulse train with an assumed blood velocity in the feeding artery of 0.3 m/s. Cerebral blood flow (CBF) was calculated using an analysis method proposed by Buxton et al^29^ with an assumed 3T arterial blood T_1_ value of 1650 ms^30^ and a 7T arterial blood T_1_ value of 2587 ms.^31^ Voxel‐wise temporal SNR (tSNR) of the pairwise‐subtracted ASL images in the central slice was calculated as the mean divided by the standard deviation of the perfusion signal across measurements. tSNR of the raw EPI series was calculated by dividing the M_0_ image by the standard deviation of control images. The mean of the control images was not used as signal in this case because presaturation was applied.

FA maps of the imaging volume were calculated based on the excitation pulse voltage and per‐Volt $B_{1}^{+}$ maps interpolated to the resolution of the imaging slices. Signal loss due to $B_{1}^{+}$ inhomogeneity in the in the gray matter mask was calculated by comparing the achieved signal and the expected signal from a uniform 90° excitation, using the following equation: signal loss = $1-\frac{S}{S_{0}}=1-\frac{\sin\left(\mathrm{FA}\right)}{\sin\left(90^{\circ }\right)}$.

## 3 RESULTS

**Figure 2:**
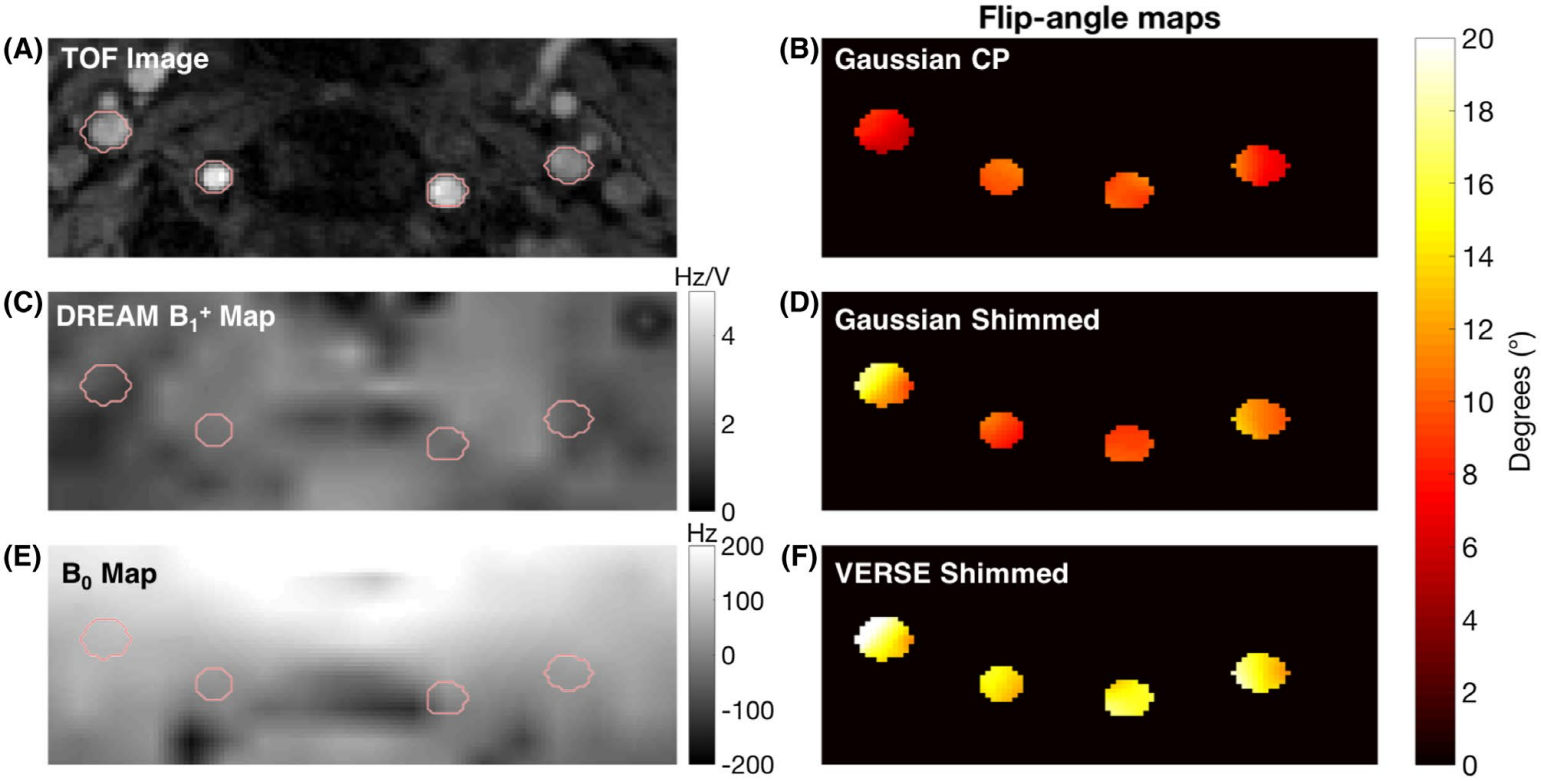
TOF image (A), γ$B_{1}^{+}$ map in Hz/V (C) and B_0_ map in Hz (E) with reduced field‐of‐view of the labeling plane. Flip‐angle (FA) maps achieved for the 3 labeling schemes (B,D,F) under identical power limits

**Figure 3:**
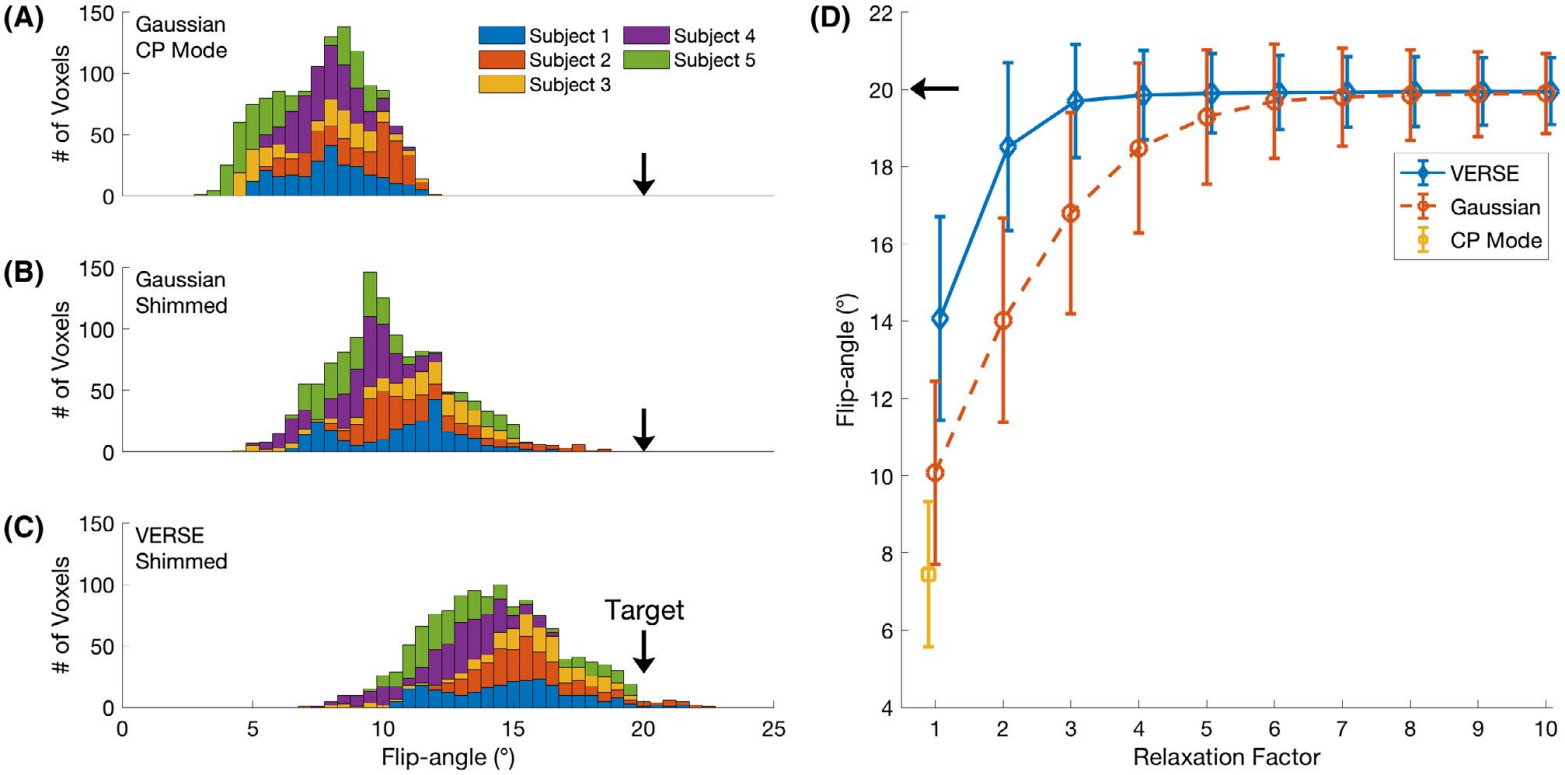
A‐C, Stacked histograms of FA achieved in the ROI of 5 subjects for each labeling scheme in simulation. D, Mean and standard deviation of the FA achieved in the ROI if the RF power constraint is relaxed

The static‐pTx RF pulse computation time was approximately 24 s. Simulation results predicted that Gaussian shimming would improve the mean FA in the ROIs across all subjects by 37% (from 7.45° to 10.17°), and that VERSE shimming would improve the mean FA by 90% (from 7.45° to 14.19°) (Figure 2, right; Figure 3A‐C). Relaxing the RF power constraint by a factor of 2 (Figure 3D) would allow for a further increase of 39% in the mean FA for the Gaussian pulse, and 31% for the VERSE pulse: a factor of 3 would enable a further increase of 67% (Gaussian pulse), and 40% (VERSE pulse). Further relaxation would only bring a marginal increase in the mean FA, but would further homogenize the FA in the ROI, resulting in a smaller standard deviation.

**Figure 4:**
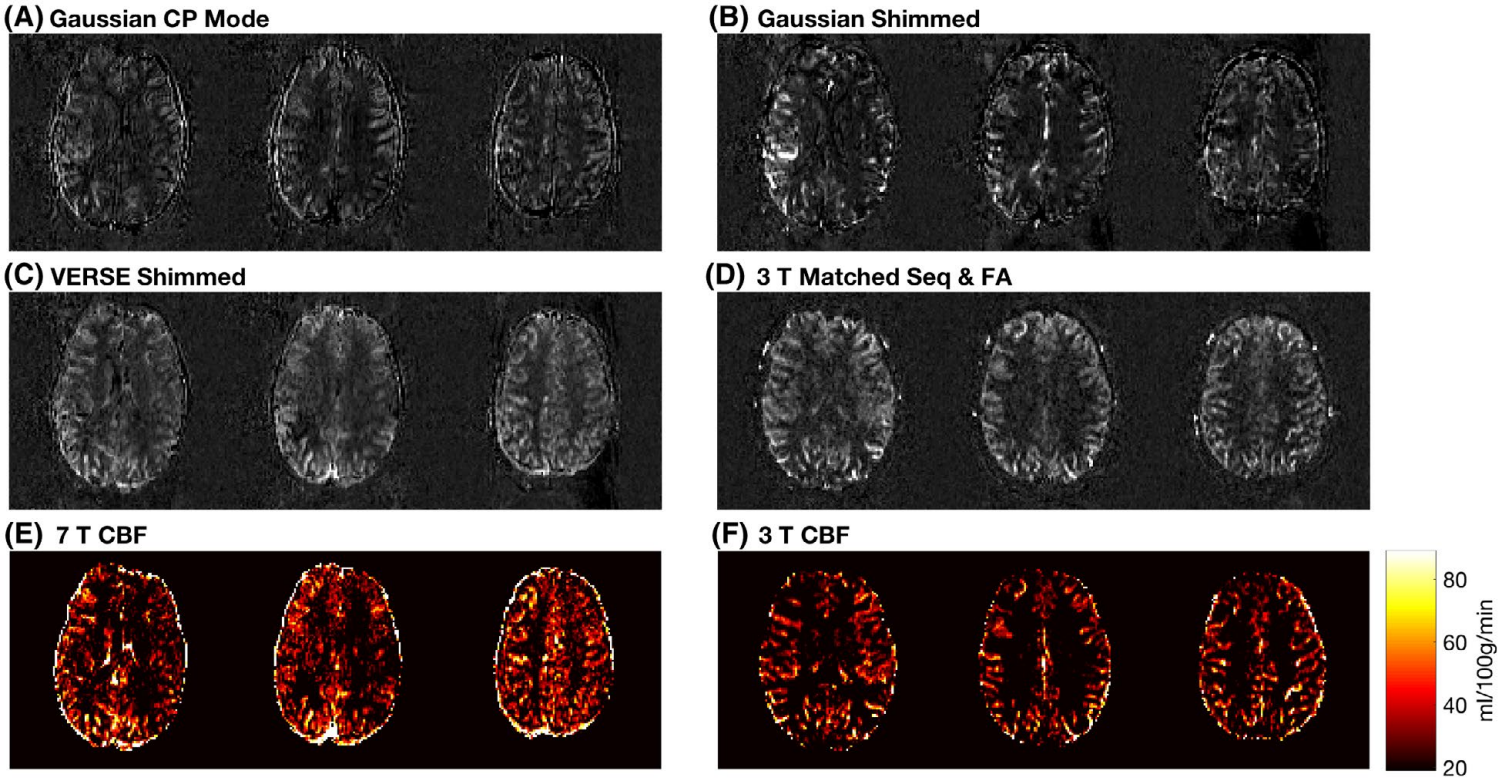
Perfusion‐weighted images and CBF maps of a representative subject. CBF maps (in mL/100 g/min) are derived from VERSE shimmed images at 7T. The 3T CBF map is from an optimized 3T sequence with 1.8 s of labeling, reduced TR of 3.2 s and double inversion background suppression

**Figure 5:**
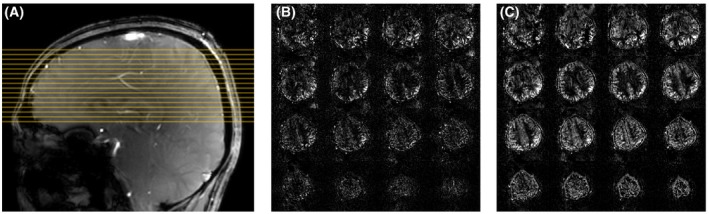
A, A sagittal localizer of subject 5 showing the slice positions of parts b and c in yellow. B, Perfusion‐weighted images of Gaussian CP mode of subject 5. c: Perfusion‐weighted images of VERSE shimming of subject 5

The quality of perfusion‐weighted images was visually improved when adopting pTx approaches over CP pulses for the labeling pulses at 7T (Figure 4). Compared with Gaussian CP mode, Gaussian pTx shimming improved the mean GM tSNR by 256% ± 316% with a peak of 697% in 1 subject. VERSE pTx shimming improved the mean GM tSNR by 375% ± 439% with a peak improvement of 1052%. The high absolute value and wide standard deviations were driven by large improvements (697.4% and 487.8%) in 2 subjects who had very low perfusion contrast under Gaussian CP mode. tSNR of the EPI series in the gray matter was 102.34 ± 57.38 for VERSE shimming at 7T, 125.22 ± 60.75 for the matched sequence with matched FA at 3T, and 200.91 ± 74.77 for the optimized 3T sequence. CBF maps generated from VERSE shimming are comparable qualitatively to those from an optimized 3T sequence (Figure 4). A whole brain perfusion‐weighted dataset is shown in Figure 5.

**Figure 6:**
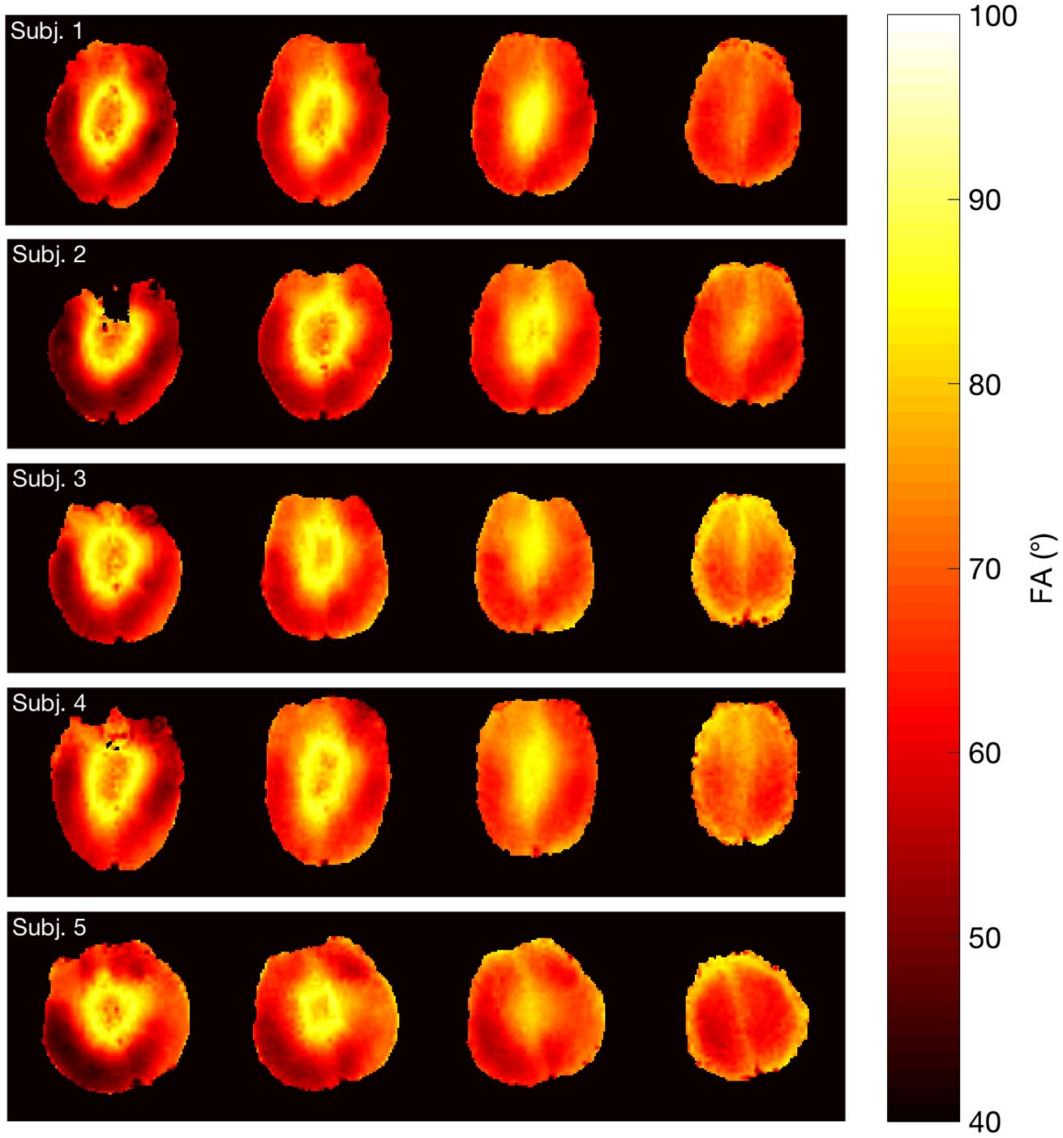
Expected FA maps of the imaging slices (slices 5, 8, 11, 14) from 5 subjects produced by the 90° excitation pulse. The FA maps were calculated based on the excitation pulse voltage and interpolated per volt $B_{1}^{+}$ maps

FA maps of the imaging volume were shown in Figure 6. The mean FA of the excitation pulse in the GM of the 5 subjects are 64.12 ± 9.55°, 63.51 ± 10.52°, 67.03 ± 10.38°, 66.77 ± 10.00°, and 63.69 ± 10.00°, respectively. Such a variation lead to an average image SNR loss of approximately 11% in the cortical gray matter.

**Figure 7:**
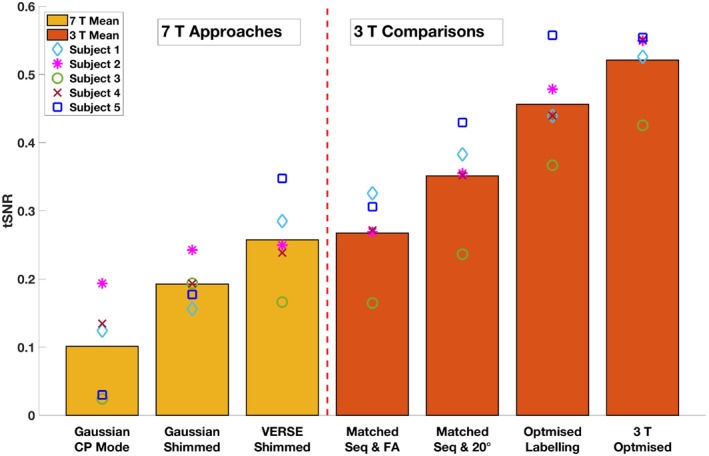
Mean GM tSNR of the perfusion‐weighted images acquired from different PCASL sequences

However, VERSE shimming did not outperform a matched 3T sequence with a matched FA in tSNR (Figure 7). Compared with VERSE shimming at 7T, a matched 3T sequence with a matched FA had a tSNR 5% higher; a matched 3T sequence with 20° FA 38% higher; a 3T sequence with optimized labeling 82% higher; and an optimized 3T sequence 110% higher.

## 4 DISCUSSION

Perfusion‐weighted images acquired at 7T using VERSE‐guided RF pTx‐shimming showed significant improvement in SNR compared with those acquired using CP mode. This is primarily due to increased FA being achieved in the labeling plane that result in a greater labeling efficiency. Because the RF power limit was included in the RF shimming calculation and all 7T approaches were power‐matched, the proposed method corrected for both $B_{1}^{+}$ and B_0_ inhomogeneity simultaneously compared with CP mode under the same RF power constraint, thus greatly improving the $B_{1}^{+}$ efficiency ($B_{1}^{+}$ per unit of RF voltage). Information on B_0_ was incorporated into the spin dynamics matrix *A*. Therefore, the proposed approach does not aim to optimize the B_0_ performance of the pulses over a range of off‐resonance values. Rather, it attempts to solve for the optimal set of shims under the off‐resonance value measured by the B_0_ map in each voxel. However, phase accrual between the PCASL labeling pulses due to B_0_ inhomogeneity was not accounted for in the proposed method. The off‐resonance correction method proposed by Berry et al^32^ could provide a complementary approach for phase accrual between pulses. The increased $B_{1}^{+}$ efficiency allows for a lower labeling plane, which in turn permits a larger imaging volume. Unlike dynamic pTx, “static” RF shimming does not involve calculation of a full RF waveform, nor optimization of the gradient trajectory. Although it is challenging to achieve a homogeneous $B_{1}^{+}$ distribution over a large field‐of‐view, static RF shimming is able to achieve more homogenous $B_{1}^{+}$ in the small ROIs used in this study with a short calculation time. Similar approaches have been introduced to improve PCASL at 7T by using either RF shimming or VERSE. Li et al proposed to improve PCASL labeling at 7T using phase‐only shimming of 8 channels.^33^ Boland et al applied VERSE on PCASL labeling pulses at 7T to reduce SAR, but no pTx strategy was adopted.^34^ Serrai et al reduced SAR by changing the sinc saturation pulses to VERSE pulses in the Q2TIPS block of a PASL sequence at 7T.^35^ Compared with PCASL, PASL at UHF suffers from a poorly defined labelled bolus and a nonuniform labeling efficiency profile across the labeling slab. However, optimizing adiabatic inversion pulses at 7T might overcome such nonuniformity. A thorough comparison between optimized PCASL and PASL at 7T is needed to evaluate whether statements established at lower fields hold true at UHF.

Despite significant improvement of tSNR for pTx‐shimming versus CP mode, VERSE pTx‐shimming at 7T did not outperform the tSNR of a matched 3T protocol with matched average FA. There are several contributing factors. First, shorter $T_{2}^{*}$ decay at 7T can lead to lower signal in the readout image.^3^ Second, our work focuses on mitigating the severe $B_{1}^{+}$ reduction that is observed at the location of the labeling plane for the type of head‐only coils typically used in UHF imaging, but significant $B_{1}^{+}$ inhomogeneity may also remain in the imaging slices. Simulation results indicated that $B_{1}^{+}$ inhomogeneity accounted for roughly an 11% loss in image SNR. Third, although the proposed method attempted to mitigate B_0_ inhomogeneity in the design of the RF pulses when the RF pulse was played out, phase accrual due to off‐resonance between neighboring RF pulses was not accounted for. Such phase accrual would lead to imperfect label and control conditions, thus reducing the perfusion signal. Fourth, although the proposed method improved the mean FA in the 4 feeding vessels, the improvement might not be uniform across the vessels. Thus, perfusion signal might differ based on the precise labeling efficiency achieved in the individual vessel from which the spins have travelled. A spatial homogeneity term could be introduced into the cost function of the RF shimming optimization. Finally, tSNR of the raw EPI series for VERSE shimming at 7T was not significantly different to a matched sequence with a matched FA at 3T. This may be due to more pronounced effects of physiological noise at 7T. Work by Triantafyllou et al showed that in fMRI time series, tSNR reaches a plateau at high image SNR in the regime where physiological noise dominates.^36^ Therefore, acquiring PCASL images with higher resolution might help better use the image SNR gain at UHF.

Adopting more complex pTx strategies for the labeling pulse train might be difficult due to the small gap between 1 labeling RF pulse and the next (1.2 ms in this study). Applying a 2‐spokes pulse is likely to produce a more homogeneous FA distribution in the ROI, but we do not expect the mean FA to improve significantly. This is because the tagging performance is primarily constrained by the low $B_{1}^{+}$ experienced in the labeling plane. Furthermore, the inversion of blood spins relies on a flow sensitive inversion process. Changing the sub‐pulse bandwidth by reducing the pulse length to adopt spokes pulses might have undesirable effects on the labeling efficiency.

All perfusion‐weighted images were acquired using the vendor’s “protected mode” SAR regime, the RF power limits of which are based on conservative local SAR estimations provided by the coil manufacturer and scanner vendor. The goal of this study was to maximize labeling efficiency under a reasonable scan time (3.5 min, TR = 6 s) and an acceptable labeling duration (1 s), although the TR could be further increased and the labeling duration further shortened to allow for a higher labeling FA to be achieved. According to the simulation results, the targeted FA of 20° across the feeding arteries in the labeling was not achieved in all subjects, primarily due to the low RF power limits. Many of the limitations of this study could be overcome by a less stringent RF power/SAR constraint. Relaxed SAR limits enabled by accurate personal electromagnetic simulations would also allow for higher labeling pulse amplitude, which could lead to further improvement in labeling efficiency. A relaxation factor of two in the power limit could be quickly realized by switching to a local SAR monitoring approach if relevant and validated electromagnetic simulations were available for the RF coil. Further relaxation by reducing vendor‐defined safety factors applied to the power measurement hardware can be expected as well. Simulations indicate that a relaxation of the RF power limit by a factor of 2 would allow a mean FA of 18.5°, whereas relaxation by a factor of 3 would allow for a mean FA of 19.7°. In addition, a longer labeling duration could also be achieved, increasing the ASL signal strength further. Moreover, additional inversion pulses for background suppression were discarded due to high SAR in this study. Under more relaxed power constraints, additional inversion pulses could be incorporated into the pulse sequence in conjunction with presaturation, potentially providing considerable improvement of background suppression to reduce physiological noise from the static tissue signal. Furthermore, more advanced pTx approaches could eliminate the need for adiabatic inversion pulses, thus reducing overall SAR.^37^

A dedicated labeling coil^8^, ^38^ could avoid $B_{1}^{+}$ attenuation in the neck seen in commercial head coils, but additional hardware would then be required. More advanced pTx strategies, such as spokes pulses^22^, ^39^ for imaging excitation, would be expected to further improve the overall perfusion image quality. However, the greatest improvements would be expected from further increased labeling efficiency/duration and an increase in the available SAR for tagging and the inclusion of a viable 7T‐compatible method for background magnetization suppression, made possible by relaxed SAR constraints.

## 5 CONCLUSION

We have demonstrated the feasibility of improving PCASL at 7T using VERSE shimming on a commercial multi‐transmit head coil under conservative SAR estimations. The tSNR achieved using VERSE shimming is comparable to that of a matched protocol at 3T. Further relaxation of SAR constraints should enable the full benefit of 7T to be realized.
